# Circular RNA profiling identifies circADAMTS13 as a miR‐484 sponge which suppresses cell proliferation in hepatocellular carcinoma

**DOI:** 10.1002/1878-0261.12424

**Published:** 2019-01-09

**Authors:** Liman Qiu, Yanbing Huang, Zhenli Li, Xiuqing Dong, Geng Chen, Haipo Xu, Yongyi Zeng, Zhixiong Cai, Xiaolong Liu, Jingfeng Liu

**Affiliations:** ^1^ The United Innovation of Mengchao Hepatobiliary Technology Key Laboratory of Fujian Province Mengchao Hepatobiliary Hospital of Fujian Medical University Fuzhou China; ^2^ The Liver Center of Fujian Province Fujian Medical University Fuzhou China; ^3^ Liver Disease Center The First Affiliated Hospital of Fujian Medical University Fuzhou China; ^4^ School of Life Science and Technology Xi'an Jiaotong University China

**Keywords:** cell proliferation, circular ADAMTS13, circular RNA, hepatocellular carcinoma, microRNA‐484

## Abstract

Circular RNA (circRNA) can participate in various biological processes, including tumorigenesis, through their microRNA response elements. Alterations in circRNA profiles during hepatocellular carcinoma (HCC) progression and their clinical significance remain unclear. Here, we present extensive analysis of circRNA profiles in tumor and matched peritumor tissues collected from 10 HCC patients, conducted to identify circRNA related to HCC progression. A total of 42 dysregulated circRNA (38 down‐regulated and 4 up‐regulated) were identified in HCC tumor tissues compared with matched peritumor tissues, revealing the heterogeneity of circRNA profiles in HCC. CircADAMTS13, derived from Exon 13–14 of the *ADAMTS13* gene, was significantly downregulated in HCC tumor tissues. Furthermore, clinicopathological analysis revealed that up‐regulation of circADAMTS13 was negatively associated with tumor size but positively associated with prognosis. In addition, overexpression of circADAMTS13 could markedly inhibit HCC cell proliferation *in vitro*. Bioinformatic analysis and luciferase reporter assays further revealed that circADAMTS13 directly interacts with microRNA (miR)‐484. Rescue experiments showed that miR‐484 mimics can reverse the tumor‐suppressing roles of circADAMTS13 in HCC. Therefore, our results demonstrated that circADAMTS13 can serve as a tumor suppressor during HCC progression via the functional pathway of sponging miR‐484.

**Abbreviations**
*ADAMTS13*: ADAM metallopeptidase with thrombospondin type 1 motif 13
AUC: area under the curve
CASP8AP2: Caspase 8‐associated protein 2
CCK‐8: cell counting kit‐8
circRNA: circular RNA
DPF3: double PHD fingers 3
GO: gene ontology
HBV: hepatitis B virus
HCC: hepatocellular carcinoma
IFN: interferon
miRNA: microRNA
ncRNA: non‐coding RNA
OS: overall survival
PI: propidium iodide
pre‐mRNA: precursor mRNA
RFS: progression‐free survival
SD: standard deviation

## 1 Introduction

Hepatocellular carcinoma (HCC) is one of the most life‐threatening and prevalent malignancies in the world (Yang and Roberts, 2010), accounting for at least 700 000 deaths worldwide every year (Marquardt *et al*., 2015). However, the mechanism of HCC progression still remains largely unclear. With the in‐depth studies of non‐coding RNA (ncRNA), accumulating evidence has revealed the vital roles of ncRNA in HCC pathogenesis (Klingenberg *et al*., 2017; Zhang *et al*., 2012). Although ncRNA do not encode proteins, they could contribute to HCC progression through regulating the expression of other genes by chromosome remodeling or transcription and post‐transcriptional processing (Wong *et al*., 2018).

Of note, circular RNA (circRNA), a peculiar class of non‐coding RNA, which are recognized as a novel category of endogenous non‐coding RNA, originate from back‐splicing of precursor mRNA (pre‐mRNA; Barrett *et al*., 2015; Chen, 2016). Unlike unidimensional RNA, circRNA formed a covalently joined continuous loop structure with neither 5′ to 3′ polarity nor the polyadenylated tail (Ashwal‐Fluss *et al*., 2014; Jeck and Sharpless, 2014; Vicens and Westhof, 2014). Thus, they are remarkably stable, highly abundant, and evolutionarily conserved (Barrett and Salzman, 2016; Memczak *et al*., 2013; Rybak‐Wolf *et al*., 2015). Natural endogenous circRNA are inherently resistant to exonucleolytic RNA decay and contain selectively conserved microRNA (miRNA) target sites, which confer the potential function of regulators or competing endogenous RNA to bind specific miRNA (e.g. miRNA sponges; Hansen *et al*., 2013; Van Peer *et al*., 2018). Indeed, several studies have discovered that specific circRNA could negatively regulate miRNA activity by competing with microRNA response elements, thus functioning as miRNA inhibitors (Hansen *et al*., 2013; Memczak *et al*., 2013). Furthermore, increasing evidence has shown that circRNA play critical roles in multiple human diseases by participating in the interaction with miRNA (Han *et al*., 2018; Zhao and Shen, 2017). Disturbing the balance of the circRNA/miRNA signal axis could lead to the onset and progression of such diseases. The most representative example is the ciRS‐7 (CDR1as)/miR‐7 signaling axis, which has been reported to regulate the progression of many diseases including neurological diseases, cardiovascular diseases, diabetes, and cancers (Han *et al*., 2018). The ciRS‐7 contains more than 60 conserved miR‐7 target sites and functions as a sponge for miR‐7; cirR‐7 overexpression could therefore result in increased expression of miR‐7‐targeted mRNA (Han *et al*., 2018). With the development of advanced sequencing techniques and bioinformatic strategies, increasing numbers of circRNA were identified during the pathophysiological processes of HCC development (Fu *et al*., 2018; Qiu *et al*., 2018). Recent studies have revealed that the circRNA‐miRNA‐mRNA axis may have crucial regulatory functions in the pathways related to HCC progression. For instance, Han *et al*. (2017) found that circMTO1 promoted p21 expression by targeting miR‐9 in HCC. Yu *et al*. (2016) confirmed that circRNA Cdr1as was significantly overexpressed in HCC tissues, enhancing HCC cell proliferation and invasion by sponging miR‐7 via promotion of CCNE1 and PIK3CD expression. The above observations supported the closely involvement of the dysregulation of circRNA and its miRNA sponge function in the pathological processes of HCC. In addition, other circRNA, including hsa_circ_0001727 and hsa_circ_0005075, have been reported to show significant potential in HCC diagnosis, prognosis, and therapy (Shang *et al*., 2016; Yao *et al*., 2017). However, similar to circRNA identified in other cancers, the circRNA involved in HCC progression should be far more abundant and the profile alteration of circRNA much more complicated. So far, a comprehensive understanding of the circRNA profiles in HCC is still lacking. Therefore, it would be extremely valuable systematically to identify and investigate the circRNA profile as well as its alterations during the pathological process of HCC, which could provide fundamental information for future in‐depth investigation of the circRNA functions.

In this study, we collected primary HCC tumor and matched peritumor tissues from 10 hepatitis B virus (HBV)‐related HCC patients to analyze the circRNA profile and its alteration during HCC progression. We screened out a significantly down‐regulated circRNA derived from exons 13 to 14 of ADAM metallopeptidase with thrombospondin type 1 motif 13 (*ADAMTS13*) and termed as circADAMTS13. Accordingly, the functions, mechanisms, and its clinical significances of this circADAMTS13 have been carefully investigated. Overall, this study has identified a novel circRNA during HCC progression and provided basic information to unravel further the functions of circRNA in HCC.

## 2 Materials and methods

### 2.1 Sample collection

All HCC primary tumor samples (*n* = 112) and matched peritumor tissue samples (*n* = 36), as well as adjacent normal tissue of hepatic hemangioma patients (*n* = 10) were collected during surgical resection of patients without preoperative treatment at Mengchao Hepatobiliary Hospital of Fujian Medical University. The 10 adjacent liver tissue samples collected from hepatic hemangioma patients were identified as normal by pathologists. The use of human tissue samples in this project was approved by the Institutional Review Board of Mengchao Hepatobiliary Hospital of Fujian Medical University and written informed consent was obtained from all patients. Clinical and pathological diagnosis of HCC patients was done following the diagnostic criteria of the American Association for the Study of Liver Diseases. The study methodologies conformed to the standards set by the Declaration of Helsinki.

### 2.2 Circular RNA sequencing and annotation

Total RNA from HCC tumor and peritumor tissue samples were extracted using TransZol reagents (TransGen Biotech, Beijing, China) according to the manufacturer's instructions. Ribosomal RNA (rRNA) were removed with a Ribo‐Zero Magnetic kit (Epicentre, Madison, WI, USA) and linear RNA was digested by RNase R (Epicentre) before construction of the RNA‐seq libraries. Briefly, ribosome and linear RNA depleted samples were fragmented and subjected to complementary (cDNA) synthesis with random hexamer primers. After repairing the ends and adding adapters, the ligated cDNA products were purified and subjected to 13–16 cycles of PCR amplification. High‐throughput sequencing was then performed by HiSeq 3000 (Illumina, San Diego, CA, USA) with a 150‐bp paired‐end run.

To identify circRNA during RNA‐seq, we first evaluated the sequencing quality of all sequencing reads and removed reads that met any of the following criteria: (1) containing adaptor sequence; (2) with > 10% ambiguous bases (N) of total read length; (3) containing > 20% low‐quality bases (base quality ≤ 20) of total read length. Qualified reads were aligned to ribosomal rRNA sequences downloaded from RNAcentral database (The RNAcentral Consortium, 2017) to remove possible rRNA inference. The unmapped reads were then aligned to UCSC human genome reference (GRCh37/hg) using TopHat2 (Kim *et al*., 2013). All the reads that could not be aligned with the genome were extracted to identify the potential back‐spliced junction reads by TopHat‐Fusion (Kim and Salzberg, 2011). The identified potential candidate reads that could be mapped to the same chromosome but in reverse order were further considered as candidate back‐spliced junction reads. After removing any circRNA with fewer than two unique back‐spliced reads in one tissue sample, the expression levels for each circRNA were calculated, according to reads Per Million mapped reads using all the junction reads within the circRNA region.

### 2.3 Cell culture

Human HCC cell line PLC/PRF/5, SK‐Hep‐1, Hep3B, hepatoblastoma cell line HepG2, as well as Human Embryonic Kidney 293 (HEK‐293) and Human Embryonic Kidney 293T (HEK‐293T) cell lines were purchased from the American Type Culture Collection (ATCC, Rockefeller, MD, USA). Human HCC cell line SMMC‐7721, Bel‐7402, and Huh7 were purchased from the Chinese Cell Bank of the Chinese Academy of Sciences (Shanghai, China). PLC/PRF/5, HepG2, SK‐Hep‐1, Hep3B, SMMC‐7721, and Huh7 cell lines were cultured in Eagle's Minimum Essential Medium (MEM; HyClone, Logan, UT, USA). Bel‐7402 cell line was cultured in RPMI‐1640 (HyClone). HEK‐293 and HEK‐293T cell lines were cultured in Dulbecco's modified Eagle medium (DMEM; HyClone). All of the cultured mediums contained 10% (v/v) FBS (Gibco, Grand Island, NY, USA). Cells were cultured in an atmosphere of 95% humidified air and 5% CO_2_ at 37 °C.

### 2.4 Establishment of the circADAMTS13 stably overexpressed cell line

Stable circADAMTS13 overexpression in the HepG2 and PLC/PRF/5 cell lines was achieved by transduction with lentivirus encoding circADAMTS13. In brief, we cloned the full‐length circADAMTS13 into the pLO‐ciR vector (Geneseed Biotech Co., Guangzhou, China), which was confirmed by Sanger sequencing. The empty vector was used as negative control (termed mock). Afterwards, HEK‐293T cells were co‐transfected with lentivirus packaging plasmid (Invitrogen, Waltham, MA, USA) and corresponding circADAMTS13/mock expression plasmids, using the Lipofectamine 3000 (Invitrogen) according to the manufacturer's protocol. Viral titers were determined using Lenti‐X GoStix (Clontech, Mountain View, CA, USA), and only lentiviruses with a minimum titer of 5 × 10^5^ infectious units (IFU)·mL^−1^ were subject to later reprogramming experiments. HepG2 and PLC/PRF/5 were co‐transduced with 5 μg·mL^−1^ Polybrene (Santa Cruz Biotechnology, Santa Cruz, CA, USA) and lentiviruses at a multiplicity of infection of 2.3. After 48 h of transfection, the transfected cells were cultured in the medium containing 2 μg·mL^−1^ puromycin (Sigma‐Aldrich, St. Louis, MO, USA) for 2 weeks to gain the positive stably transfected clone with overexpression of circADAMTS13. The selected cell lines were further used for *in vitro* experiments.

### 2.5 Cell proliferation assay

Cell proliferation assays were carried out using the cell counting kit‐8 (CCK‐8) assay and colony formation assay. For CCK‐8 assay, cells were seeded in 96‐well plates at a density of 5 × 10^3^ cells per well and incubated at 37 °C in 5% CO_2_. An aliquot of 10%(v/v) CCK‐8 reagent (TransGen Biotech) was added to each well and incubated at 37 °C for 2 h. The absorbance (A) was measured at the wavelength of 450 nm according to the manufacturer's protocol (Molecular Devices LLC, Sunnyvale, CA, USA). Samples were then measured every 24 h after 4 days of culture. The experiments were repeated independently for five times. For Colony formation assay, cells were seeded in six‐well plates at a density of 5 × 10^2^ cells per well and incubated at 37 °C in 5% CO_2_ for 2 weeks. After that, formed colonies were fixed with 4% paraformaldehyde (Beyotime Biotechnology, Shanghai, China) for 20 min and then stained with 0.1% crystal violet (Beyotime Biotechnology) for 10 min. A number of clones were imaged and counted under microscope. The experiments were repeated independently three times.

### 2.6 Flow cytometry analysis of cell apoptosis

After being serum‐starved for 24 h, the cells were divided into two groups. One group underwent H_2_O_2_ treatment (400 μm) for 12 h to induce cell apoptosis. Afterwards, 0.5–1 × 10^6^ cells were collected by centrifugation, and incubated with Annexin V/propidium iodide (PI) using an Annexin V‐FITC/PI Cell Apoptosis Detection Kit (TransGen Biotech) according to the manufacturer's instructions. The cells were then analyzed by flow cytometry analysis after incubation for 15 min in dark. The early apoptotic cells were stained with Annexin V alone, whereas necrotic and late apoptotic cells were stained with both Annexin V and PI.

### 2.7 Actinomycin D and RNase R treatment

For actinomycin D treatment, 2 μg·mL^−1^ actinomycin D (Sigma‐Aldrich) or DMSO (Sigma‐Aldrich) as a control was added to the cell culture medium. For RNase R treatment, 2 μg of total RNA was incubated 30 min at 37 °C with or without 3 U·mg^−1^ of RNase R (Epicentre Technologies).

### 2.8 qRT‐PCR

Total RNA were isolated from fresh‐frozen tissues and cultured cells using TransZol reagents (TransGen Biotech). Reverse transcription was carried out using a Transcriptor First Strand cDNA Synthesis Kit (Roche, Indianapolis, IN, USA) according to the manufacturer's instructions. The divergent primers for circADAMTS13, circDPF3, and circCASP8AP2 were designed to determine the abundance of circRNA. Bulge‐Loop™ hsa‐miR‐484 qRT‐PCR Primer Set (RiboBio, Guangzhou, China) and U6 snRNA qPCR Primer Set (RiboBio) were used for amplification of miR‐484 and U6, respectively. The other primers are listed in Table S1. For qRT‐PCR analysis, aliquots of double‐stranded cDNA were amplified using SYBR Premix Ex Taq II (Takara, Dalian, China). The relative gene expression was normalized to the geometric mean of the housekeeping gene *18SrRNA* and then calculated according to the Livak method (∆∆*C*_t_; Livak and Schmittgen, 2001). The experiments were independently repeated three times.

### 2.9 Luciferase reporter assay

The interaction between circADAMTS13 and miR484 was measured using the pMIR‐REPORTTM system. The full length of circADAMTS13 (termed circADAMTS13‐WT) and mutated circADAMTS13 (mutated in each miR‐484 target site of the circADAMTS13 sequence, termed circADAMTS13‐Mut) were inserted into pMIR‐REPORT vector (Ambion, Waltham, MA, USA). Luciferase reporter assays were performed according to the manufacturer's instructions. Briefly, HEK‐293T cells were transfected with corresponding pMIR‐REPORT constructs, β‐Gal and miR‐484 mimic (or scramble control) using Lipofectamine 3000 in a 24‐well plate. The cells were harvested after transfection for 48 h and the luciferase activity was measured using the BrightGloTM Luciferase Assay System (Promega, Madison, WI, USA) according to the manufacturer's instructions. β‐Gal activities were measured as internal control. Data were obtained by normalization of β‐Gal activity to luciferase activity. The bars represent the mean ± SEM from three independent experiments.

### 2.10 RNA sequencing, gene ontology, and pathway analyses

Total RNA were isolated from ex‐circADAMTS13‐PLC/PRF/5 and mock‐PLC/PRF/5 cells using the Trizol Reagent (TransGen Biotech). The samples with RNA integrity number scores higher than eight were then subjected to sequencing library preparation. The acquired sequencing libraries were sequenced on an Illumina HiSeq 4000 platform with a 150‐bp paired‐end run by Novogene Bioinformatics Institute (Beijing, China) after enrichment (13–16 cycles of PCR amplification) and quality control. The gene expression levels in each sample were estimated according to fragments per kilo‐base of exon per million fragments mapped. The gene expression comparison was conducted using Student's *t* test and transcripts with a *P *<* *0.05 were considered differentially expressed in different groups of cells. To understand the functional roles of these differentially expressed genes, ConsensusPathDB (http://cpdb.molgen.mpg.de/CPDB) was used to conduct gene ontology (GO) enrichment analysis (GO level 2 categories) and pathway enrichment analysis (pathways defined by KEGG and Reactome databases). Pathways with *P *<* *0.05 were considered significantly enriched.

### 2.11 Statistical analysis

Statistical analysis was performed using spss software (version 19.0; IBM Corp., Armonk, NY, USA) and R software (version 3.4.3; R Foundation for Statistical Computing, Vienna, Austria). The *t* test was applied to analyze the statistical significance between two groups. The statistical significance between more than two groups was analyzed by one‐way variance (ANOVA) analysis, followed by the Bonferroni correction for multiple comparisons. Categorical data were analyzed by Fisher's exact test between circADAMTS13 expression and clinicopathological features. The linear dependence was analyzed by Spearman's correlation. The differences in progression‐free survival (RFS) and overall survival (OS) of HCC patients with low or high circADAMTS13 expression levels were estimated using the Kaplan‐Meier method, and the log‐rank test was performed to examine the significance. The expression of circADAMTS13 was considered to be either low (*n* = 76) or high (*n* = 26) according to the cutoff value, which was defined as the third quartile of circADAMTS13 expression level. *P *<* *0.05 was considered to be statistically significant.

## 3 Results

### 3.1 Identification of the circRNA profile in HCC tumor and matched peritumor tissues

**Figure 1:**
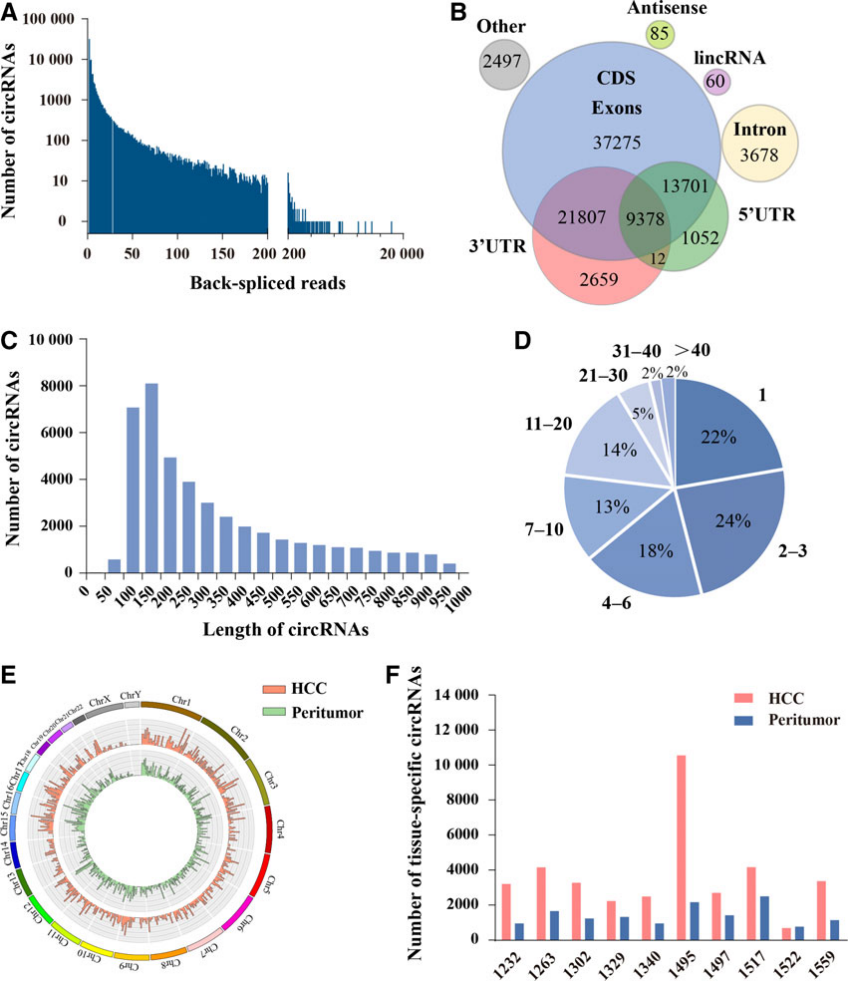
Identification of circRNA in HCC tumor and matched peritumor tissues. (A) The number of circRNA and junction reads identified in 10 pairs of HCC tumor and matched peritumor tissues. (B) Genomic origin of identified circRNA. (C) The length distribution of circRNA. (D) Number of circRNA produced from one gene. (E) The distribution of identified circRNA in chromosomes. The orange and green bars represent the location of circRNA within different chromosomes in HCC tumor and matched peritumor tissues, respectively. (F) Number of circRNA specifically expressed in either 10 HCC tumor tissues or their matched peritumor tissues. The numbers in the horizontal axis represent the sample numbers.

To characterize the circRNA profile involved in HCC progression, RNA sequencing was performed for ribosomal and linear‐depleted RNA extracted from 10 pairs of HCC tumor and matched peritumor tissues (Table S2). We obtained an average of 53 million (range from 42 to 63) mapping reads for each sample. The bioinformatic tool ‘TopHat2’ was applied to identify circRNA in both tumor and matched peritumor tissues based on the anchor alignment of unmapped reads. As shown in the Fig. 1A, a total of 92 204 circRNA were identified in these 20 tissue samples with more than two unique back‐spliced reads (Table S3). Among them, 20 404 circRNA (23.13%) have been identified in other studies in circBase (Glazar *et al*., 2014), and the other 71 800 circRNA (77.87%) are novel. We further annotated these identified candidates using the Ensemble database (Kersey *et al*., 2018) and found that 81.9% of them were identified in exons, the others originating from noncoding regions: on average, 3.7% in the intronic region, 2.7% in the 3′UTR region, 1% in the 5′UTR region, etc. (Fig. 1B). Consistent with previous studies, the results showed that the length of identified circRNA was mostly around 100–450 nucleotides (Fig. 1C). Moreover, analysis of the circRNA numbers from host genes revealed that an average of 8 circRNA (ranging from 1 to 175) were produced by one host gene (Fig. 1D). Furthermore, based on the gene location, these circRNA were evenly distributed on each chromosome, showing no obvious difference between HCC tumor and matched peritumor tissue samples (Fig. 1E). Subsequently, by extracting tissue‐specific and non‐specific circRNA, we found 35 302 circRNA (38.29%) specifically expressed in either HCC tumor or matched peritumor tissue samples. The numbers of tissue‐specific circRNA for each sample are shown in Fig. 1F, demonstrating the high heterogeneity of circRNA formation among individuals.

### 3.2 CircRNA specifically associated with HCC progression

**Figure 2:**
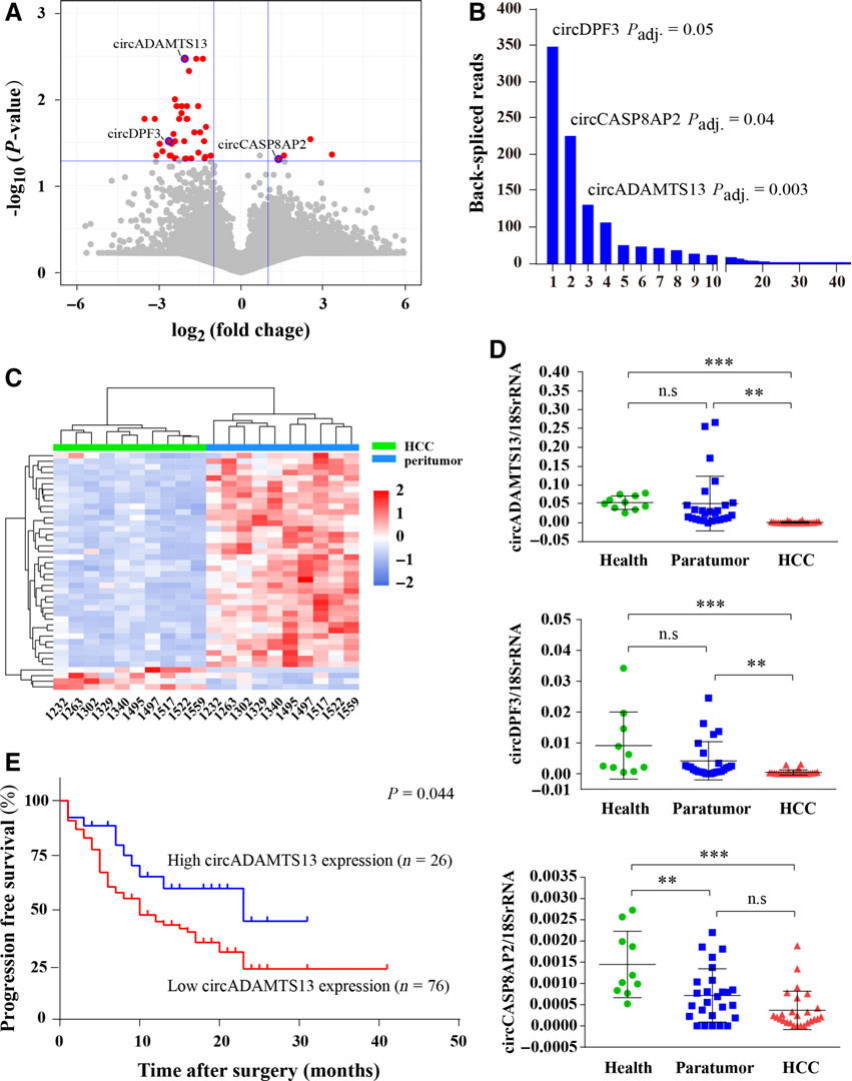
Dysregulated circRNA profiles in HCC. (A) Volcano plot of the differences in circRNA abundance between HCC tumor tissues and matched peritumor tissues. The *x*‐axis specifies the negative logarithm to base 2 of the fold changes, and the *y*‐axis specifies the negative logarithm to base 10 of the adjusted *P* values calculated by limma. Blue vertical and horizontal lines reflect the filtering criteria (fold change ≥ 2.0 and *P* ≤ 0.05). The red points in plot represent the dysregulated circRNA with statistical significance. (B) Back‐splicing reads of the 42 dysregulated circRNA. The vertical axis represents the back‐splicing reads of circRNAs, and the horizontal axis represents the circRNA display order through back‐splicing reads. (C) Clustered heat map of the 42 dysregulated circRNA between HCC tumor and peritumor tissues. Each column represents one tissue sample and each row indicates a transcript. (D) Relative expression of the circADAMTS13, circDPF3, and circCASP8AP2 in 10 healthy liver tissues, as well as 26 pairs of HCC tumor tissues and their matched peritumor tissues measured by qRT‐PCR. The statistical significance among groups was analyzed by ANOVA analysis followed by the Bonferroni correction. **P <* *0.01; ***P < 0.001; n.s., no statistical significance. Error bars indicate standard deviation. (E) Kaplan–Meier analysis of the association between circADAMTS13 expression level and the RFS time of patients with HCC. Those patients with high circADAMTS13 expression (*n* = 26) had significantly longeer RFS time than those with low circADAMTS13 expression (*n* = 76).

To identify further the dysregulation of circRNA during HCC progression, bioconductor ‘limma’ was used to compare circRNA expression values between HCC tumor and matched peritumor tissues. A total of 42 circRNA, which were dysregulated in all HCC tumor tissue samples when compared with matched peritumor tissues (fold change ≥ 2 and *P *<* *0.05), were shown in the volcano plot (Fig. 2A, Table S4). Consistent with previous studies (Chen *et al*., 2016; Fu *et al*., 2018), among the 42 dysregulated circRNA, most of them (38/42) were downregulated in HCC tissue samples. These results suggested that the fluctuation of the expression levels of these circRNA might be involved in the development of HCC. The back‐spliced reads and expression patterns of those dysregulated circRNA were also displayed in Fig. 2B and C, respectively. We selected three circRNA with most significant change in expression level and a high number of back‐spliced reads to validate their expression in further 26 HCC patients (Table S5), including circADAMTS13 (hsa_circ:chr9:136302869‐136303486), circDPF3 (hsa_circ:chr14:73181131‐73198642), and circCASP8AP2 (hsa_circ:chr6:90556281‐90566918). We subsequently amplified those three circRNA in HCC tumor and matched peritumor tissue samples using their own divergent primers. As shown in Fig. 2D, circADAMTS13 and circDPF3 were significantly down‐regulated in HCC tumor tissue samples when compared with matched peritumor tissue samples (*P *=* *0.0016 and *P *=* *0.0058, respectively), which were consistent with the sequencing data in our study. Compared with healthy liver tissue samples, the expression levels of these three circRNA were significantly down‐regulated in HCC tumor tissue samples (Fig. 2D) and could clearly differentiate HCC tumor tissues from healthy liver tissues [Fig. S1A; circADAMTS13 area under the curve (AUC) = 0.987; circDPF3 AUC = 0.946; circCASP8AP2 AUC = 0.923]. Moreover, the expression levels of circADAMTS13 and circDPF3 showed no significant difference in healthy liver tissues and peritumor tissues, implying that circADAMTS13 and circDPF3 might specifically indicate the occurrence of HCC. Taken together, these results suggest that circADAMTS13 and circDPF3 may participate in HCC progression.

To investigate the role of circADAMTS13 and circDPF3 in HCC progression, we next analyzed the association between the expression levels of these two circRNA and the clinical characteristics in 102 HCC patients. As summarized in Table 1, low expression of circADAMTS13 in HCC tumor tissues was associated with the absence of liver cirrhosis (*P *=* *0.023, Fig. S1B), larger tumor size (*P *=* *0.001, S1C,D), and more severe BCLC stage (*P *=* *0.030, Fig. S1E). There was no significant correlation of circDPF3 with these investigated clinical characteristics (data not shown). Furthermore, Kaplan–Meier analysis showed that the HCC patients with low circADAMTS13 expression had a significant shorter RFS time (*P *=* *0.044, Fig. 2E), but did not show significant OS (*P *=* *0.071, Fig. S1F). However, the expression of circDPF3 could not clearly distinguish the clinical outcomes of these HCC patients (*P *=* *0.257, data no shown). Taking together, these data suggest that down‐regulation of circADAMTS13 expression in HCC is clearly associated with a poor patient prognosis. Therefore, circADAMTS13 might represent a novel potential prognosis biomarker for HCC, and it was selected for further mechanism study.

**Table 1:** Associations between the expression levels of circADAMTS13 and the clinicopathological characteristics of 102 HCC patients

Clinical characteristics	CircADAMTS13 expression		χ^2^ value	*P* value^a^
	Low (*n* = 76)	High (*n* = 26)		
Sex				
Male	70	25	0.497	0.426
Female	6	1		
Age, years				
< 55	40	17	1.278	0.184
≥ 55	36	9		
AFP, ng·mL^−1^				
< 400	53	20	0.492	0.332
≥ 400	23	6		
HBV DNA, cps·mL^−1^				
< 500	28	13	1.395	0.171
≥ 500	48	13		
Cirrhosis				
Absent	12	0	4.653	0.023*
Present	64	26		
Tumor number				
Single	66	22	0.081	0.502
Multiple	10	4		
Maximal tumor size, cm				
< 5	34	21	10.123	0.001***
≥ 5	42	5		
Tumor metastasis				
Absent	73	25	0.001	0.732
Present	3	1		
Tumor differentiation				
I–II	18	10	2.124	0.116
III–IV	58	16		
Microvascular invasion				
Absent	37	16	1.282	0.183
Present	39	10		
TNM				
I–II	55	22	1.570	0.162
III–IV	21	4		
HCC stage, BCLC				
0–A	31	17	4.704	0.030*
B–C	45	9		

^a^Fisher's exact test was applied to analyze the statistical significance. *P <* *0.05; ***P <* *0.001.

### 3.3 Characteristics of the circular RNA circADAMTS13

**Figure 3:**
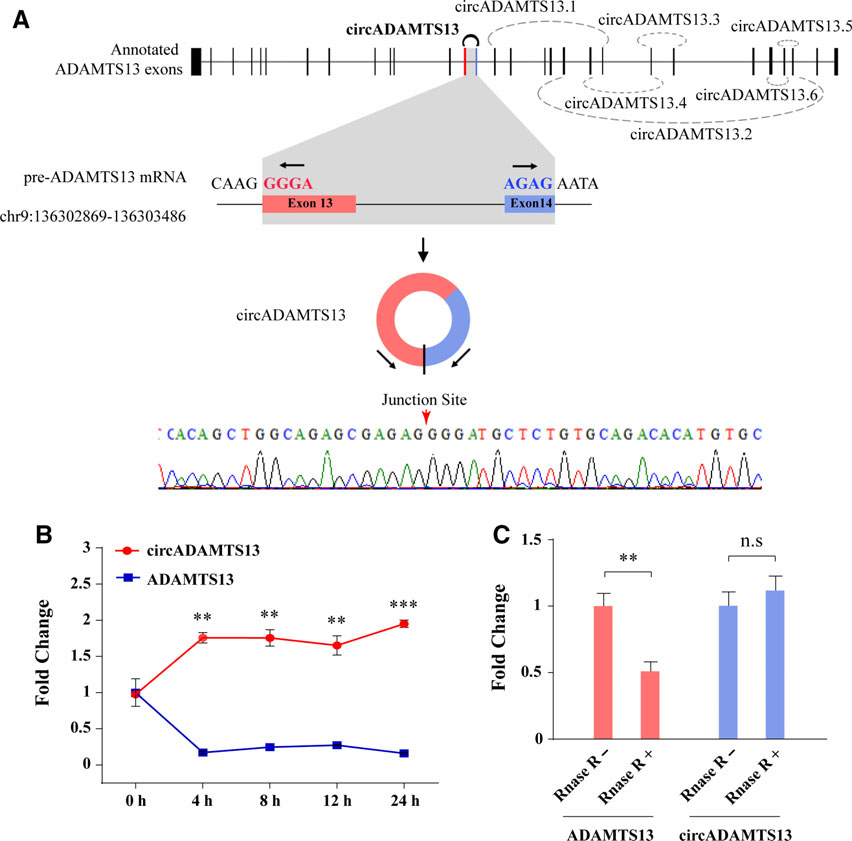
The characteristics of the circular RNA circADAMTS13. (A) The genomic loci of all the identified circRNA produced by ADAMTS13 gene, and the splicing of circADAMTS13. The presence of circADAMTS13 was validated by RT‐PCR followed by Sanger sequencing. (B) qRT‐PCR analysis of the abundance of circADAMTS13 and ADAMTS13 mRNA in HEK‐293 cells treated with Actinomycin D at the indicated time points. Values are presented as geometric means ± 95% confidence interval (error bar). (C) qRT‐PCR analysis of the abundance of circADAMTS13 and ADAMTS13 mRNA in HEK‐293 cells treated with RNase R. Rnase R^−^, non‐Rnase R‐treated group; Rnase R^–^, Rnase R‐treated group. Error bars indicate standard deviation. The amount of circADAMTS13 and ADAMTS13 mRNA in (B, C) was normalized to the value measured in the non‐treated group. The statistical significance between two groups was analyzed by *t* test. **P <* *0.01; ***P <* *0.001; n.s., no statistical significance.

In our study, seven circRNA isoforms were identified in the *ADMATS13* gene locus: circADAMTS13, circADAMTS13.1, circADAMTS13.2, circADAMTS13.3, circADAMTS13.4, circADAMTS13.5, circADAMTS13.6 (Fig. 3A). Interestingly, only circADAMTS13 isoform has high back‐spliced unique junction reads (83) when compared with other isoforms (circADAMTS13.1: 4 reads, circADAMTS13.2: 4 reads, circADAMTS13.5: 4 reads, circADAMTS13.4: 4 reads, circADAMTS13.6: 4 reads, circADAMTS13.3: 7 reads). The expected sequences of circADAMTS13 were amplified using divergent primers and confirmed by Sanger sequencing (Fig. 3A). Previous studies have shown that circRNA were more stable and not easily degraded by RNase R due to their unique formation of non‐sequential back‐splicing of pre‐mRNA transcripts (Memczak *et al*., 2013; Suzuki and Tsukahara, 2014). We therefore next examined the stability of circADAMTS13 by actinomycin D (an inhibitor of transcription) intervention and RNase R treatment. The qRT‐PCR assays revealed that circADAMTS13 was highly stable with a half‐life > 24 h, whereas linear ADAMTS13 mRNA exhibited a half‐life of < 4 h (Fig. 3B). Digestion resistance experiments further confirmed that circADAMTS13 could be resistant to RNase R, whereas linear ADAMTS13 mRNA was easily degraded (Fig. 3C). Taken together, our results showed the high structural stability of circADAMTS13, which was consistent with the general characteristics of circRNA.

### 3.4 CircADAMTS13 could suppress HCC cell proliferation

**Figure 4:**
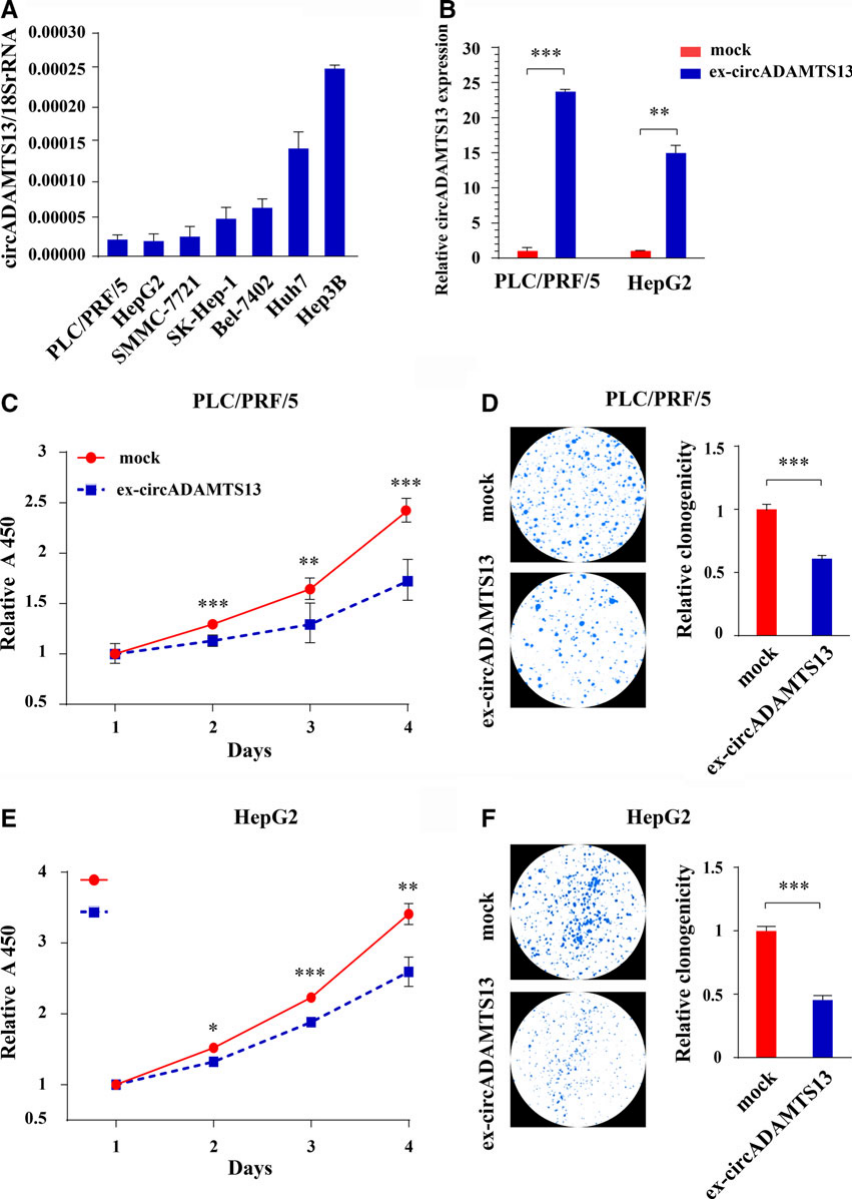
Circular ADAMTS13 suppresses cell proliferation of HCC cells. (A) qRT‐PCR analysis of the abundance of circADAMTS13 in tumor cell lines of liver origin (PLC/PRF/5, HepG2, SMMC‐7721, SK‐Hep‐1, Bel‐7402, Huh7, Hep3B). (B) Overexpression of circADAMTS13 was validated by qRT‐PCR in PLC/PRF/5 and HepG2 cells. The circADAMTS13 expression level in mock was normalized to 1. Error bars indicate standard deviation (SD). (C,E) Proliferation assessment of PLC/PRF/5 (C) and HepG2 (E) cells with stable expressing circADAMTS13 by CCK‐8 assay. The relative A450 value of cells grown in mock group for 1 day was normalized to 1. Values were represented as geometric means ± 95% confidence interval (error bar). (D,F) Left panel: colony numbers of PLC/PRF/5 (D) and HepG2 (F) cells with stable expression of circADAMTS13 studied by colony formation assay. Right panel: quantitative analysis of left panel. The relative clonogenicity in the mock group was normalized to 1. Error bars indicate SD. The *t* test was applied to analyze the statistical significance between two groups. *P <* *0.05; **P <* *0.01; ***P <* *0.001.

To explore the functional role of circADAMTS13 in HCC progression, the expression of circADAMTS13 was first evaluated in six tumor cell lines of liver origin (PLC/PRF/5, HepG2, SMMC‐7721, SK‐Hep‐1, Bel‐7402, Huh7, Hep3B). As shown in Fig. 4A, PLC/PRF/5 and HepG2 expressed relative low levels of circADAMTS13. We therefore selected PLC/PRF/5 and HepG2 cells to establish stable circADAMTS13 overexpression cell model by lentivirus transduction. The overexpression efficiency of circADAMTS13 in transfected PLC/PRF/5 and HepG2 cells was verified by qRT‐PCR. As shown in Fig. 4B, the qRT‐PCR results showed that circADAMTS13 was ~ 23 times overexpressed in ex‐circADAMTS13‐PLC/PRF/5 cells and ~ 15 times overexpressed in ex‐circADAMTS13‐HepG2 cells.

We further investigated the effects of circADAMTS13 overexpression on cell proliferation, migration, and invasion. CCK‐8 assay and colony formation assay both showed that circADAMTS13 overexpression strongly suppressed cell proliferation of PLC/PRF/5 and HepG2 cells (Fig. 4C–F). In addition, circADAMTS13 overexpression increased the percentage of apoptotic cells (Annexin V^+^/PI^+^ and Annexin V^+^/PI^−^ cells) compared with mock cells (*P *<* *0.05) according to the flow cytometric analysis, suggesting the regulatory effects of circADAMTS13 on cell apoptosis (Fig. S2A,B); however, the migration and invasion ability of HCC cells was not significantly changed (data not shown). These results suggest that circADAMTS13 plays an important role in HCC cell proliferation inhibition and apoptosis, but does not affect the cell migration and invasion, consistent with previous clinical results.

### 3.5 CircADAMTS13 serves as a sponge for oncogenic miR‐484

**Figure 5:**
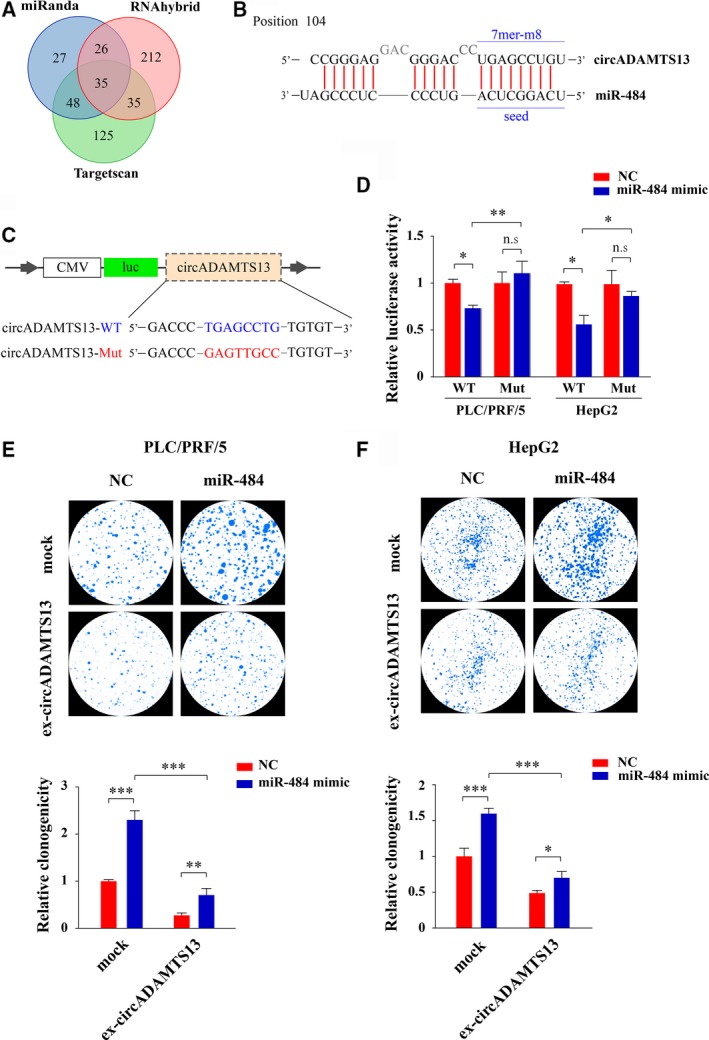
Circular ADAMTS13 serves as a sponge for oncogenic miR‐484. (A) The overlapping of potential circADAMTS13 binding miRNA, which were predicted by three independent miRNA databases (miRanda, TargetScan, and RNAhybrid). (B) Schematic presentation of the putative binding sites of miR‐484 with respect to circADAMTS13. (C) Schematic illustration of the circADAMTS13 wild‐type (WT) and mutant (Mut) luciferase reporter vectors. (D) The relative luciferase activities of PLC/PRF/5 and HepG2 cells which were co‐transfected with miR‐484 mimics, or NC and pMIR‐circADAMTS13‐WT, or pMIR‐circADAMTS13‐Mut luciferase reporter vectors. The relative luciferase activities of WT group treated with miR‐NC were normalized to 1. (E,F) Top panel: the numbers of colonies of PLC/PRF/5 (E) and HepG2 (F) cells co‐transfected with miR‐484 mimic or miR‐NC that were assessed using colony formation assay. Bottom panel: quantitative analysis of top panel. The relative clonogenicity in the mock group treated with miR‐NC was normalized to 1. The statistical significance among groups was analyzed by ANOVA analysis followed by the Bonferroni correction. *P <* *0.05; **P <* *0.01; ***P <* *0.001; n.s., no statistical significance. Error bars indicate standard deviation.

We sought to grasp the potential molecular mechanism of circADAMTS13 during HCC progression. Since it has been reported that the function of cancer‐related circRNA was mainly as miRNA sponges to bind functional miRNA and then regulate cancer‐related signaling pathways (Yang *et al*., 2017), we used three independent miRNA databases (miRanda: http://www.microrna.org/microrna/home.do, Targetscan: http://www.targetscan.org/, and RNAhybrid: http://bibiserv.techfak.uni-bielefeld.de/rnahybrid/) to predict the circADAMTS13 binding miRNA. The Venn diagram in Fig. 5A shows that a total of 35 miRNA were predicated to be the possible down‐stream targets of circADAMTS13. Among all of these candidate miRNA, miR‐484, a newly discovered key molecule for the malignant transformation of HCC (Yang *et al*., 2016), has strong binding ability (Mfe = −38.3 kcal·mol^−1^, *P *=* *0.001) to circADAMTS13. The bioinformatics prediction search (by Targetscan) also showed that circADAMTS13 could harbor miR‐484 by miRNA seed sequence matching (Fig. 5B). These *in silico* analysis results supported the hypothesis that circADAMTS13 might serve as a binding platform for oncogenic miR‐484. Therefore, we further detected the expression levels of miR‐484 in different cell lines and tissue samples. As shown in Fig. S3A, the cell lines with low circADAMTS13 expression generally had higher miR‐484 expression. Moreover, the miR‐484 expression was significantly up‐regulated in HCC tumor tissues when compared with matched peritumor tissues and healthy liver tissues (*P *=* *0.0028 and *P *<* *0.0254, respectively; Fig. S3B). Not surprisingly, the expression level of circADMATS13 was negatively correlated with the expression level of miR‐484 (*r* = −0.456, *P *=* *0.043), indicating that circADAMTS13 might regulate miR‐484 expression by directly sponging miR‐484 (Fig. S3C). To validate this hypothesis, we performed a luciferase screening for miR‐484. Here, the circADAMTS13‐WT and circADAMTS13‐Mut were inserted downstream of the luciferase reporter (Fig. 5C). The miR‐484 mimic was then co‐transfected with the luciferase reporters into PLC/PRF/5 and HepG2 cells. Compared with the control miRNA (termed NC), miR‐484 was able to reduce the luciferase reporter activities of circADAMTS13 in PLC/PRF/5 and HepG2 at ~ 30% and ~ 40%, respectively, but miR‐484 had no significant effect on luciferase activity in the mutated target sites of circADAMTS13 (Fig. 5D). These results suggested that circADAMTS13 could directly bind to miR‐484.

To determine whether the circADAMTS13 could affect cell proliferation in HCC via sponging miR‐484, we performed colony formation assays using PLC/PRF/5 and HepG2 cells transfected with circADAMTS13 overexpressing vector (or mock), together with the miR‐484 or NC. As shown in Fig. 5E,F, the results demonstrated that circADAMTS13 overexpression remarkably suppressed HCC cell clonogenicity, whereas up‐regulation of miR‐484 in the meantime could significantly rescue the suppression effects of circADAMTS13. Overall, these results demonstrated that circADAMTS13 acted as a tumor suppressant by regulating the activity of miR‐484.

## 4 Discussion

Hepatocellular carcinoma is a highly heterogeneous malignancy resulting from intricate genetic and epigenetic alterations. Nowadays, limited treatment options and poor therapy responses are still major challenges for improving the clinical outcomes worldwide. Further screening potential diagnostic/prognostic biomarkers and therapeutic targets, as well as in‐depth investigation of possible molecular mechanisms are crucial for optimizing HCC treatment. Recently, noncoding RNA (ncRNA) have become very important targets and a hot area of HCC study. Several studies have already suggested that tumor‐specific ncRNA could be used for survival prediction and therapeutic intervention (Klingenberg *et al*., 2017; Quagliata *et al*., 2014; Wang *et al*., 2014). Among those HCC‐associated ncRNA, circRNA have potentially important roles in the carcinogenesis and progression of HCC (Fu *et al*., 2018; Han *et al*., 2018). Several circRNA, such as circMTO1 and cSMARCA5, have already been suggested to play important roles during HCC progression by acting as sponges of oncogenic miRNA, and may be potential targets for HCC treatment (Han *et al*., 2017; Yu *et al*., 2018). In our study, we also identified a circRNA named circADAMTS13, which played important roles in the cell proliferation process of HCC cells, through comprehensive investigation of the circRNA profiles of 10 pairs of HCC tumor and matched peritumor tissues, as well as the associated patients’ clinical‐pathological characteristics.

Circular ADAMTS13 has been previously identified via deep sequencing in human brain samples, glioma tissues, and lung fibroblast cell line AG04450 (Rybak‐Wolf *et al*., 2015; Salzman *et al*., 2013; Song *et al*., 2016), but its function remains largely unknown. Interestingly, the large amount of complementary Alu repeats included in the circADAMTS13 pre‐mRNA indicated that the formation of circADAMTS13 may result from the direct back‐splicing mechanism with intronic RNA pairings. In this study, we showed that the expression level of circADAMTS13 was significantly correlated with the BCLC stage of patients. Since BCLC staging has been widely applied in the evaluation of HCC patients to decide on the recommended treatment (Marrero *et al*., 2018), the expression level of circADAMTS13 might be effective in reflecting the tumor status of patients and thus might serve as a biomarker for evaluating the tumor burden of patients. Furthermore, our current study showed that circADAMTS13 was remarkably down‐regulated in HCC tumor tissues and could serve as a suppressor of cell proliferation by sponging miR‐484 in HCC cells. Of note, the circRNA/miRNA axis has been reported recently to regulate gene expressions at both transcription and post transcription levels (Chen *et al*., 2016; Han *et al*., 2018). We therefore further performed RNA sequencing analysis of ex‐circADAMTS13 and mock‐PLC/PRF/5 cells to investigate the downstream dysregulated genes. In total, 288 differently expressed genes were identified, 39 of which overlapped with the predicted target genes of miR‐484 (prediction tool: miRPathDB, https://mpd.bioinf.uni-sb.de/; Table S6). As shown in Fig. S4, 39 potential downstream genes of circADAMTS13/miR‐484 signal axis were mainly involved in binding and biological activities: catalytic, receptor, signal transducer, and transporter activity. Moreover, the biological process analysis revealed that these potential downstream genes of the circADAMTS13/miR‐484 signal axis mainly participated in metabolic process and cellular process, nicely in line with the remarkably dysregulated metabolism and cellular processes of cancer cells. It is well known that metabolic networks played key roles in biological regulation, thus controlling the cellular processes in different time scales (Green *et al*., 2014). However, cancer cells fundamentally altered cellular metabolism, which could directly lead to the dysregulation of cell process and eventually change cell fate (Dumas *et al*., 2017; Martinez‐Outschoorn *et al*., 2017). Based on the above observations, circADAMTS13 is likely to regulate HCC metabolism and cellular processes by affecting downstream genes of miR‐484, thereby inhibiting the proliferation of HCC.

Of note, miR‐484 was reported to be a crucial trigger for the precancerous process of HCC. Previous studies showed that overexpression of miR‐484 promoted hepatocyte transformation and hepatoma development in a hepatocyte orthotopic transplantation model and a high‐fat‐diet‐induced tumor model (Tessitore *et al*., 2016; Yang *et al*., 2016). Although interferon β (IFN‐β) could serve as a regulator for miR‐484 expression, the direct upstream effect of miR‐484 remained unclear (Lechel and Gougelet, 2016). Undoubtedly, our study provided new insights into the regulatory mechanisms of miR‐484 in HCC. What's more important, since circADAMTS13 could directly inhibit the activity of oncogenic miR‐484 and further exert its influence in HCC cell proliferation, the circADAMTS13 might therefore be considered a potential therapeutic target for HCC in the future. There were some encouraging studies regarding the circRNA‐based therapies. For instance, Jost *et al*. (2018) successfully constructed artificial circRNA containing an array of miRNA‐22‐binding sites, to inhibit the replication and translation of hepatitis C virus by efficiently sponging miRNA‐22. Hence, the application of circADAMTS13 in HCC therapy is also worth considering. However, additional studies should be conducted to explore the actual tumor suppression function as well as the synthesis and degradation mechanism of circADAMTS13 *in vivo*.

## 5 Conclusion

Through a comprehensive analysis of circRNA profiles during HCC progression, our study provides new understanding of tumor heterogeneity at the circRNA level. Moreover, we showed that circADAMTS13 was specifically down‐regulated in HCC tumor tissues and could serve as a key modulator of HCC cell growth by efficiently sponging miR‐484. Accordingly, our findings highlighted that circADAMTS13 might have potential values for understanding the complicated molecular mechanisms of HCC progression and improving the treatment of HCC.

## Conflict of interest

The authors declare no conflict of interest.

## Author contributions

Study concept and design: LQ, ZC, XL, and JL. Acquisition of data: LQ, YH, ZL, XD, GC, HX, and YZ. Analysis and interpretation of data: LQ, YH, and ZL. Statistical analysis: LQ, XD, and GC. Drafting of the manuscript: LQ and ZL. Critical revision of the manuscript: ZC, XL, and JL. Obtaining funding: XL and JL. Study supervision: ZC, XL and JL.

## Supporting information


**Fig. S1.** Circular RNA specifically associated with HCC progression. (A) ROC curves as well as the AUC (Area Under the Curve) values of 3 candidate circRNAs for differentiating HCC tumor tissues from healthy liver tissues. (B) Relative expression of the circADAMTS13 in HCC tumor tissues with or without cirrhosis. (C) Relative expression of the circADAMTS13 in HCC tumor tissues with tumor size of <5 cm and ≥5 cm. (D) Scatter diagram of the spearman correlation between circADAMTS13 expression level and tumor size. (E) Relative expression of the circADAMTS13 in HCC tumor tissues of patients at 0‐A stage and B‐C stage in BCLC staging. (F) Kaplan‐Meier analysis of the association between circADAMTS13 expression level and the overall survival time of patients with HCC. The statistical significance between two groups was analyzed by T‐test. *p<0.05; ***p<0.001. Error bars indicate SD.

**Fig. S2.** Effect of circADAMTS13 on cell apoptosis by Annexin V/PI staining. Representative dot plots of Annexin V/PI staining of mock and ex‐circADAMTS13 cells with or without H_2_O_2_ treatment (400 μM, 12 h) performed in PLC/PRF/5 (A) and HepG2 (B) cells. Annexin V‐/PI‐ (lower left) cells represented survival cells, Annexin V+/PI‐ (lower right) cells were defined as early apoptotic cells, Annexin V+/PI+ (upper right) cells were recognized as late apoptotic cells, Annexin V+/PI‐ (upper left) cells were considered as necrotic cells. The cell apoptotic rates were summarized (right panel). The statistical significance between two groups was analyzed by T‐test. *p<0.05; **p<0.01; n.s represents no statistical significance. Error bars indicate SD.

**Fig. S3.** Correlation between expression of circADAMTS13 and miR‐484. (A) The expression level of circADAMTS13 and miR‐484 in tumor cell lines of liver origin. (B) The expression level of miR‐484 in healthy liver tissues, HCC tumor tissues and their matched peritumor tissues measured by qRT‐PCR. The statistical significance among groups was analyzed by ANOVA analysis followed by the Bonferroni correction. *p<0.05; **p<0.01; n.s represents no statistical significance. Error bars indicate SD. (C) Scatter diagram showed the spearman correlation between the expression level of circADAMTS13 and miR‐484.

**Fig. S4.** GO analysis of the potential downstream genes of circADAMTS13/miR‐484 signaling axis.

**Table S1.** Primers for qRT‐PCR used in this study.

**Table S2.** Clinical information of 10 HCC patient samples used for sequencing.

**Table S3.** The circRNA identified by RNA‐seq in 10 pairs of human HCC tumor and matched peritumor tissues.
**Table S4.** The expression level of 42 dysregulated circRNA in 10 pairs of human HCC tumor and matched peritumor tissues.

**Table S5.** Clinical data on 26 HCC patient samples for the validation of circRNA expression.
**Table S6.** Data on 39 potential downstream genes of circADAMTS13/miR‐484 signaling axis.
