# Supplementary figures and images for: Circular RNA profiling identifies circADAMTS13 as a miR‐484 sponge which suppresses cell proliferation in hepatocellular carcinoma
Source: Mol Oncol. 2019 Jan 9;13(2):441–55. doi: 10.1002/1878-0261.12424 (PMC6360375; doi:10.1002/1878-0261.12424)

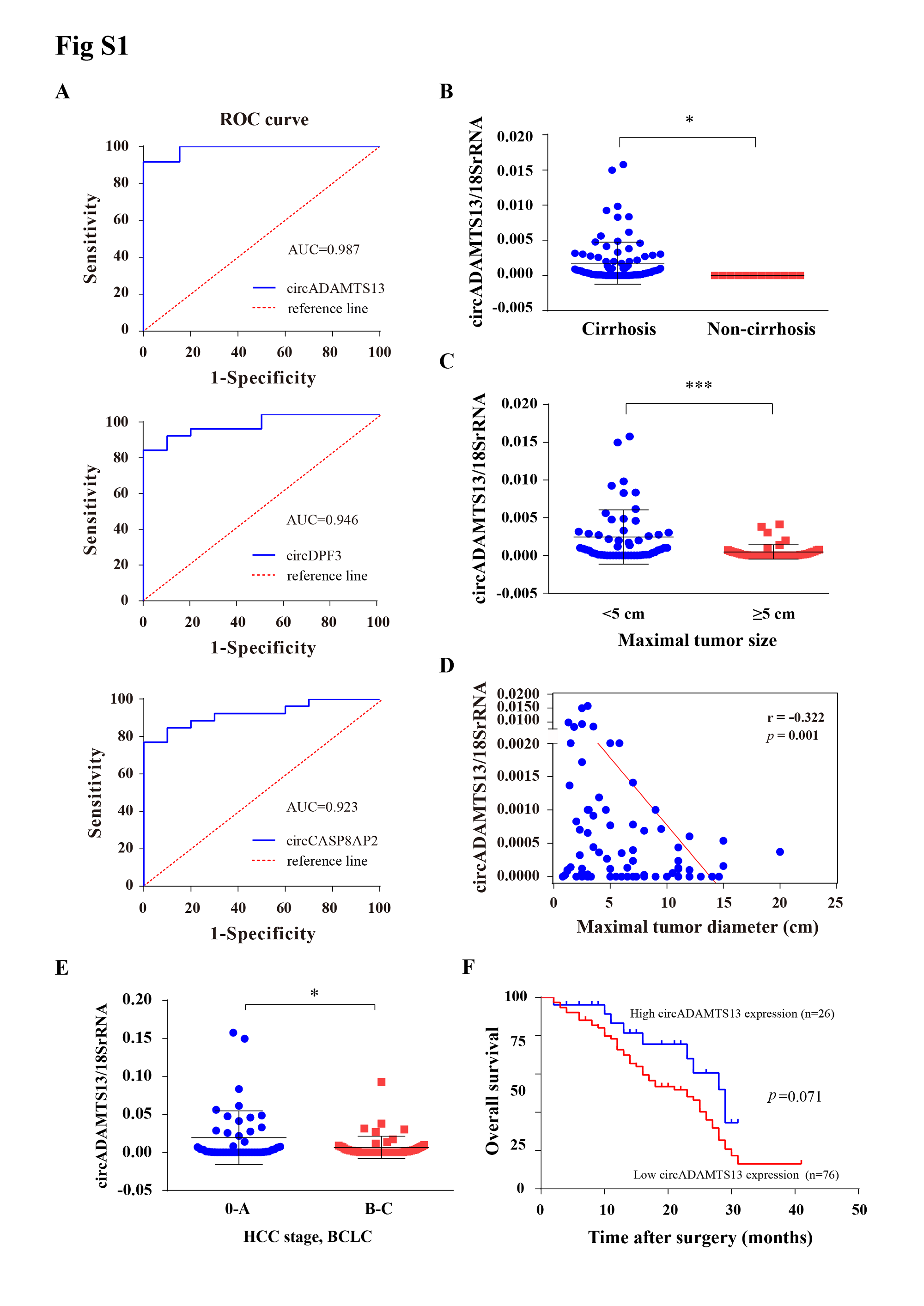

Supplement: Supplementary file 1 — Fig. S1. Circular RNA specifically associated with HCC progression. (A) ROC curves as well as the AUC (Area Under the Curve) values of 3 candidate circRNAs for differentiating HCC tumor tissues from healthy liver tissues. (B) Relative expression of the circADAMTS13 in HCC tumor tissues with or without cirrhosis. (C) Relative expression of the circADAMTS13 in HCC tumor tissues with tumor size of <5 cm and ≥5 cm. (D) Scatter diagram of the spearman correlation between circADAMTS13 expression level and tumor size. (E) Relative expression of the circADAMTS13 in HCC tumor tissues of patients at 0‐A stage and B‐C stage in BCLC staging. (F) Kaplan‐Meier analysis of the association between circADAMTS13 expression level and the overall survival time of patients with HCC. The statistical significance between two groups was analyzed by T‐test. *p<0.05; ***p<0.001. Error bars indicate SD. [file MOL2-13-441-s001.tif]

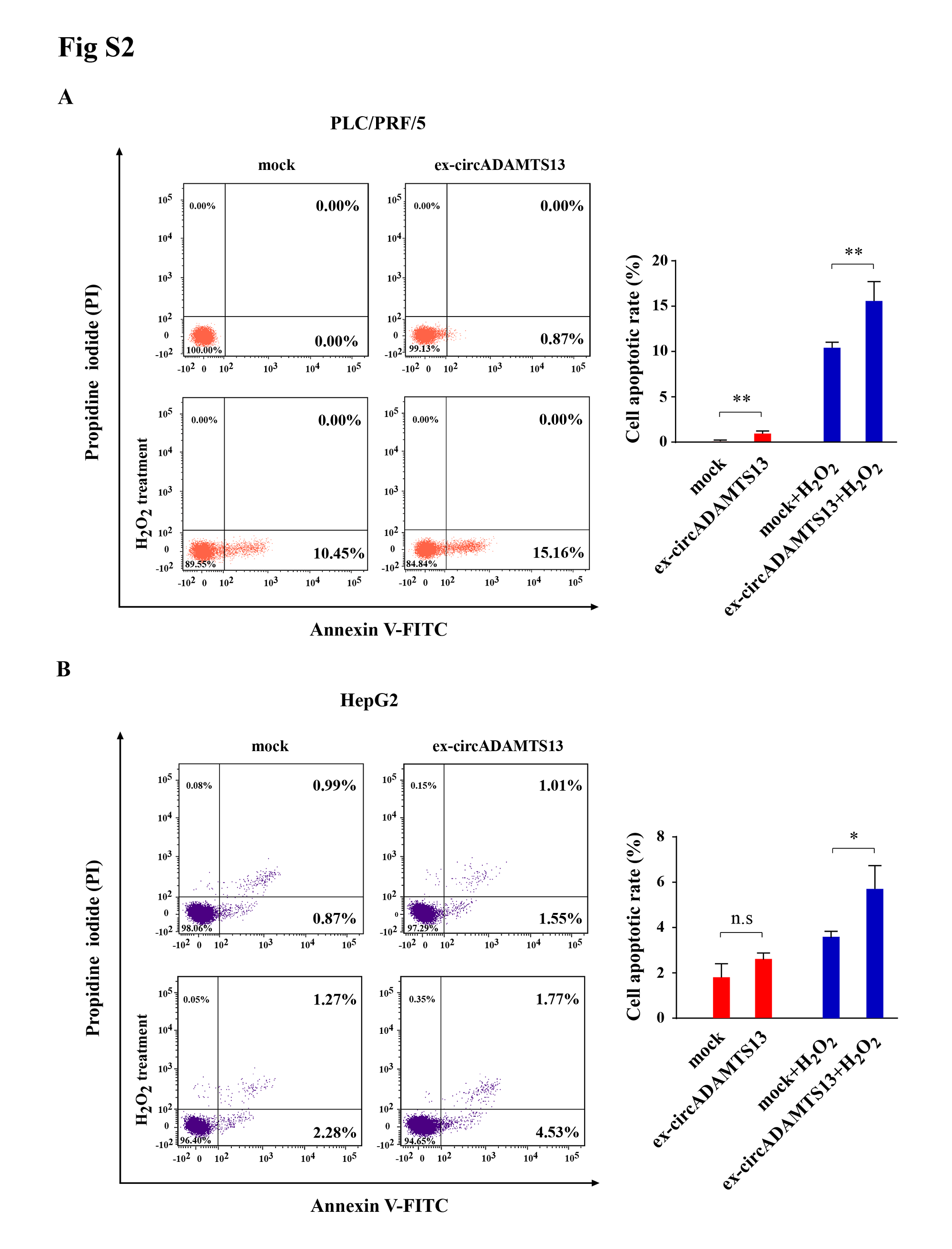

Supplement: Supplementary file 2 — Fig. S2. Effect of circADAMTS13 on cell apoptosis by Annexin V/PI staining. Representative dot plots of Annexin V/PI staining of mock and ex‐circADAMTS13 cells with or without H2O2 treatment (400 μM, 12 h) performed in PLC/PRF/5 (A) and HepG2 (B) cells. Annexin V‐/PI‐ (lower left) cells represented survival cells, Annexin V+/PI‐ (lower right) cells were defined as early apoptotic cells, Annexin V+/PI+ (upper right) cells were recognized as late apoptotic cells, Annexin V+/PI‐ (upper left) cells were considered as necrotic cells. The cell apoptotic rates were summarized (right panel). The statistical significance between two groups was analyzed by T‐test. *p<0.05; **p<0.01; n.s represents no statistical significance. Error bars indicate SD. [file MOL2-13-441-s002.tif]

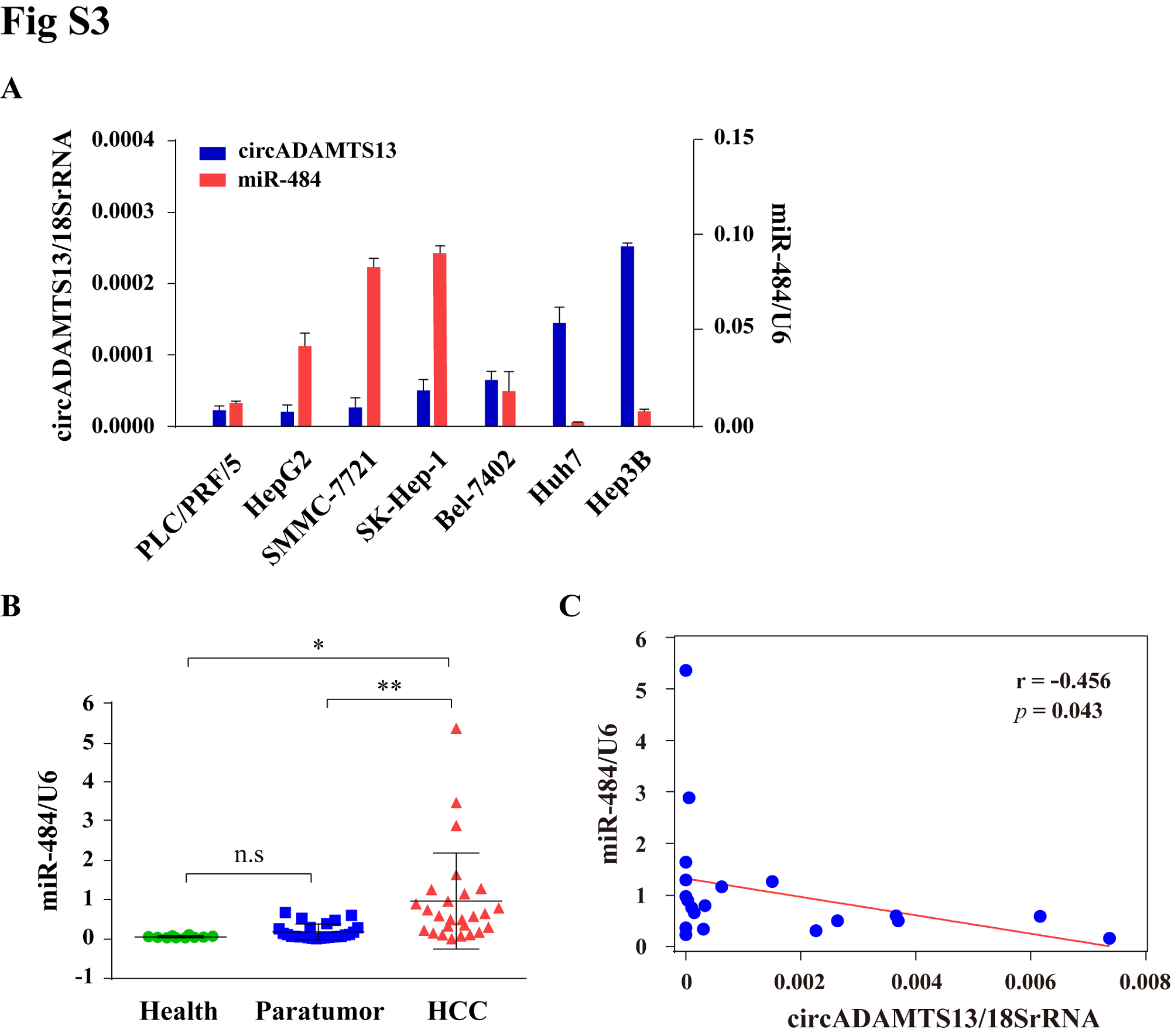

Supplement: Supplementary file 3 — Fig. S3. Correlation between expression of circADAMTS13 and miR‐484. (A) The expression level of circADAMTS13 and miR‐484 in tumor cell lines of liver origin. (B) The expression level of miR‐484 in healthy liver tissues, HCC tumor tissues and their matched peritumor tissues measured by qRT‐PCR. The statistical significance among groups was analyzed by ANOVA analysis followed by the Bonferroni correction. *p<0.05; **p<0.01; n.s represents no statistical significance. Error bars indicate SD. (C) Scatter diagram showed the spearman correlation between the expression level of circADAMTS13 and miR‐484. [file MOL2-13-441-s003.tif]

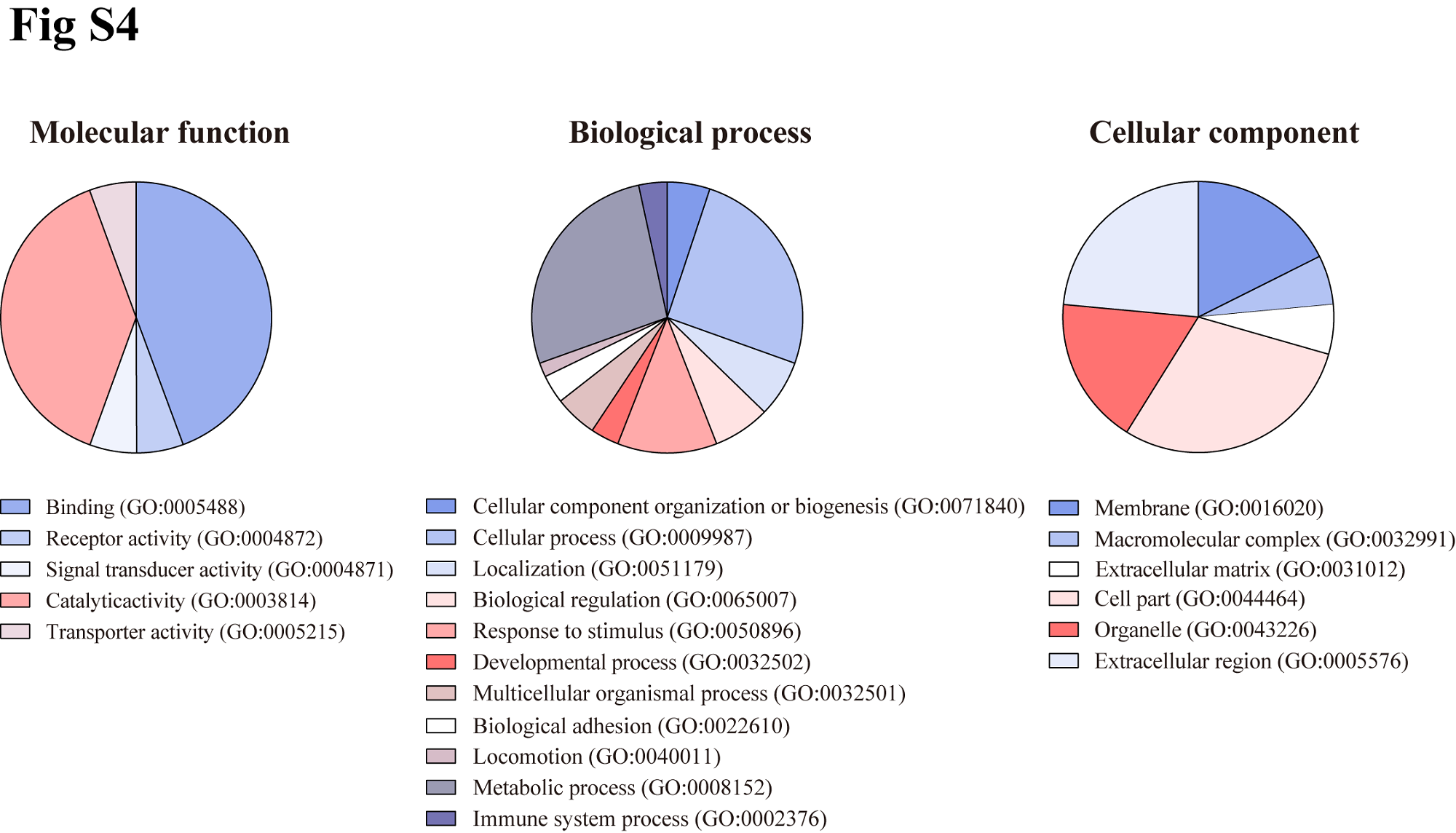

Supplement: Supplementary file 4 — Fig. S4. GO analysis of the potential downstream genes of circADAMTS13/miR‐484 signaling axis. [file MOL2-13-441-s004.tif]
