# Supplementary material for: Circular RNA profiling identifies circADAMTS13 as a miR‐484 sponge which suppresses cell proliferation in hepatocellular carcinoma
Source: Mol Oncol. 2019 Jan 9;13(2):441–55. doi: 10.1002/1878-0261.12424 (PMC6360375; doi:10.1002/1878-0261.12424)
Supplement: Supplementary file 5 — Table S1. Primers for qRT‐PCR used in this study. [file MOL2-13-441-s005.docx]

| **Primer name** | **Sequence (5'** → **3')** |
| --- | --- |
| circADAMTS13-F | ACGGAAGGGCTCTTTCACA |
| circADAMTS13-R | GGGCCACTTGGCATACA |
| circDPF3-F | TCCCAAGCGAAAGAACAGGA |
| circDPF3-R | CCACCTTCTTCTCAACCCCC |
| circCASP8AP2-F | CTGCCAGAGTGGAAATAAACCG |
| circCASP8AP2-R | TTGTTTCTGCTGCCATGATCC |
| mADAMTSM3-F | GAGACAGCTTCCTCGATGGG |
| mADAMTSM3-R | CTGTGAAAGAGCCCTTCCGT |
| 18SrRNA-F | TTCGGAACTGAGGCCATGAT |
| 18SrRNA-R | CGAACCTCCGACTTTCGTTCT |

**Table S1. Primers for qRT-PCR used in this study**
