# Supplementary material for: Circular RNA profiling identifies circADAMTS13 as a miR‐484 sponge which suppresses cell proliferation in hepatocellular carcinoma
Source: Mol Oncol. 2019 Jan 9;13(2):441–55. doi: 10.1002/1878-0261.12424 (PMC6360375; doi:10.1002/1878-0261.12424)
Supplement: Supplementary file 6 — Table S2. Clinical information of 10 HCC patient samples used for sequencing. [file MOL2-13-441-s006.docx]

**Table S2**. Clinical information of 10 HCC patient samples used for sequencing

| **Patient ID** | **Gender** | **Age** | **HBV-DNA (IU/ml)** | **Serum AFP (ng/ml)** | **Maximal tumor diameter (cm)** | **Number of tumors** | **Portal invasion** | **Cirrhosis** | **HCC stage** | |
| --- | --- | --- | --- | --- | --- | --- | --- | --- | --- | --- |
|  |  |  |  |  |  |  |  |  | **TNM** | **BCLC** |
| 1232 | Male | 50 | 1.90×10^4^ | 11.20 | 4 | 1 | No | Yes | T1N0M0 | A |
| 1263 | Male | 60 | 1.20×10^6^ | 14.04 | 5 | 1 | No | Yes | T1N0M0 | A |
| 1302 | Male | 55 | <500 | 2.11 | 3.5 | 1 | No | Yes | T1N0M0 | A |
| 1329 | Male | 44 | 1.73×10^2^ | 8.23 | 3 | 1 | No | Yes | T1N0M0 | A |
| 1340 | Male | 49 | 7.40×10^2^ | 106.70 | 2.2 | 1 | No | Yes | T1N0M0 | A |
| 1495 | Male | 57 | <500 | 9.47 | 6 | 1 | No | Yes | T1N0M0 | A |
| 1497 | Male | 57 | 6.20×10^5^ | 17.70 | 7 | 1 | Yes | Yes | T2N0M0 | C |
| 1517 | Female | 43 | 6.50×10^5^ | 73.18 | 5.5 | 1 | No | Yes | T1N0M0 | A |
| 1522 | Male | 69 | <500 | 4.26 | 4.5 | 1 | No | Yes | T1N0M0 | A |
| 1559 | Male | 58 | <500 | 30.46 | 2.3 | 1 | No | Yes | T1N0M0 | A |
