# Supplementary material for: Circular RNA profiling identifies circADAMTS13 as a miR‐484 sponge which suppresses cell proliferation in hepatocellular carcinoma
Source: Mol Oncol. 2019 Jan 9;13(2):441–55. doi: 10.1002/1878-0261.12424 (PMC6360375; doi:10.1002/1878-0261.12424)
Supplement: Supplementary file 8 — Table S5. Clinical data on 26 HCC patient samples for the validation of circRNA expression. Table S6. Data on 39 potential downstream genes of circADAMTS13/miR‐484 signaling axis. [file MOL2-13-441-s008.docx]

**Table S5**. Clinical information of 26 HCC patient samples for the validation of circRNA expression

| **Patient** | **Gender** | **Age** | **HBV-DNA (IU/ml)** | **Serum AFP (ng/ml)** | **Maximal tumor diameter (cm)** | **Number of tumors** | **Portal invasion** | **Cirrhosis** | **HCC stage** | |
| --- | --- | --- | --- | --- | --- | --- | --- | --- | --- | --- |
|  |  |  |  |  |  |  |  |  | **TNM** | **BCLC** |
| 1 | Male | 30 | 4.92×10^7^ | 271.40 | 7 | 1 | No | Yes | T1N0M0 | A |
| 2 | Male | 64 | <500 | 3.00 | 3.5 | 1 | No | Yes | T1N0M0 | A |
| 3 | Male | 57 | <500 | >2000 | 11 | 1 | No | No | T1N0M0 | A |
| 4 | Male | 63 | 1.92×10^4^ | 265.00 | 2 | 1 | No | Yes | T1N0M0 | 0 |
| 5 | Male | 40 | <500 | 1.78 | 3 | 1 | No | Yes | T1N0M0 | A |
| 6 | Male | 44 | 6.10×10^4^ | 96.50 | 8 | 1 | No | Yes | T1N0M0 | A |
| 7 | Male | 64 | 1.96×10^3^ | 43.80 | 4 | 1 | No | Yes | T1N0M0 | A |
| 8 | Male | 62 | <500 | 3.45 | 11 | 1 | No | No | T1N0M0 | A |
| 9 | Male | 56 | 6.73×10^2^ | 14.00 | 1.2 | 1 | No | Yes | T1N0M0 | 0 |
| 10 | Male | 53 | <500 | 5.40 | 1 | 1 | No | Yes | T1N0M0 | 0 |
| 11 | Male | 58 | 2.08×10^2^ | 12.00 | 5.8 | 1 | No | Yes | T1N0M0 | A |
| 12 | Male | 61 | <500 | 1.10 | 5 | 1 | No | Yes | T1N0M0 | A |
| 13 | Male | 66 | <500 | 4.70 | 3 | 1 | No | Yes | T1N0M0 | A |
| 14 | Male | 73 | <500 | 3.30 | 2.5 | 1 | No | Yes | T1N0M0 | A |
| 15 | Female | 60 | 1.60×10^5^ | 165.00 | 5 | 1 | Yes | Yes | T2N0M0 | C |
| 16 | Female | 57 | <500 | 885.10 | 3 | 1 | No | Yes | T2N0M0 | A |
| 17 | Male | 45 | 2.50×10^7^ | >2000 | 11 | 1 | No | Yes | T2N0M0 | A |
| 18 | Male | 76 | <500 | 3.27 | 3.1 | 1 | No | No | T1N0M0 | A |
| 19 | Male | 41 | 5.30×10^3^ | 60.85 | 14 | 1 | No | No | T1N0M0 | A |
| 20 | Male | 56 | <500 | 2.95 | 2.3 | 1 | No | Yes | T1N0M0 | A |
| 21 | Male | 31 | 25000000 | >80000 | 14 | 1 | Yes | No | T2N0M0 | C |
| 22 | Male | 46 | <500 | 9.70 | 2.5 | 1 | No | Yes | T1N0M0 | A |
| 23 | Male | 58 | 169000 | 4.15 | 4 | 1 | No | Yes | T1N0M0 | A |
| 24 | Male | 64 | 1960 | 43.80 | 4 | 1 | No | Yes | T1N0M0 | A |
| 25 | Male | 45 | 4700000 | 6.30 | 1.5 | 1 | No | Yes | T1N0M0 | 0 |
| 26 | Female | 62 | 12900 | >2000 | 2 | 1 | No | Yes | T1N0M0 | 0 |
